# A novel rapid quantitative method reveals stathmin‐1 as a promising marker for esophageal squamous cell carcinoma

**DOI:** 10.1002/cam4.1449

**Published:** 2018-03-25

**Authors:** Lu Yan, Xiu Dong, Jiajia Gao, Fang Liu, Lanping Zhou, Yulin Sun, Xiaohang Zhao

**Affiliations:** ^1^ State Key Laboratory of Molecular Oncology National Cancer Center/Cancer Hospital Chinese Academy of Medical Sciences & Peking Union Medical College Beijing China

**Keywords:** AlphaLISA, biomarker, esophageal squamous cell carcinoma, exosomes, stathmin‐1

## Abstract

Stathmin‐1 is a microtubule depolymerization protein that regulates cell division, growth, migration, and invasion. Overexpression of stathmin‐1 has been observed to be associated with metastasis, poor prognosis, and chemoresistance in various human cancers. Our previous studies found that serum stathmin‐1 was significantly elevated in patients with esophageal squamous cell carcinoma (ESCC) by ELISAs. Here, we constructed high‐affinity monoclonal antibodies and then developed a competitive AlphaLISA for rapid, accurate quantitation of stathmin‐1 in serum. Compared to ELISA, our homogeneous AlphaLISA showed better sensitivity and accuracy, a lower limit of detection, and a wider linear range. The measurements of nearly 1000 clinical samples showed that serum stathmin‐1 level increased dramatically in patients with squamous cell carcinoma (SCC), especially in ESCC, with a sensitivity and a specificity of 81% and 94%, respectively. Even for early stage ESCC, stathmin‐1 achieved an area under the receiver operating characteristic curve (AUC) of 0.88. Meanwhile, raised concentrations of stathmin‐1 were associated with lymph node metastasis and advanced cancer stage. Notably, various types of SCC showed significantly higher AUCs in serum stathmin‐1 detection compared to adenocarcinoma. Furthermore, we confirmed that stathmin‐1 was enriched in the oncogenic exosomes, which can explain the reason why it enters into the blood to serve as a tumor surrogate. In conclusion, this large‐scale and systematic study of serum stathmin‐1 measured by our newly established AlphaLISA showed that stathmin‐1 is a very promising diagnostic and predictive marker for SCC in the clinic, especially for ESCC.

## Introduction

Esophageal squamous cell carcinoma (ESCC) is the most common type of esophageal cancer in Asia and Africa. Due to its asymptomatic nature in the early stage, most cases are diagnosed at advanced stages with poor prognosis. In China, it remains the fourth leading cause of cancer‐related death 1. At present, early detection of ESCC still depends on invasive endoscopic examinations 2. There are no available serum markers for ESCC in the clinic. Squamous cell carcinoma antigen (SCC‐Ag) and Cyfra21‐1 showed low sensitivity in ESCC patients 3. Therefore, there is an urgent need to discover specific diagnostic markers for ESCC.

Stathmin‐1 (STMN1) is a microtubule regulatory protein. It prevents polymerization of tubulin heterodimers to destabilize the microtubule cytoskeleton. Thus, it plays important roles in the control of cellular division and cell cycle progression 4, 5. Our previous studies found that stathmin‐1 was overexpressed in the tissues and sera from patients with ESCC, and it promoted invasion and metastasis of ESCC cells via activation of the integrinα5β1/FAK/ERK pathway 6, 7. The increased expression of stathmin‐1 in tissues was associated with poor prognosis and chemoresistance in a variety of human malignancies, including ESCC, ovarian cancer, prostate cancer, gastric cancer, non‐small cell lung cancer, endometrial cancer, oral cancer, bladder cancer 8, 9, 10, 11, 12, 13, 14, 15, 16, 17, 18.

Notably, our and other groups found that stathmin‐1 levels in sera were elevated in patients with ESCC, non‐small cell lung cancer, and bladder cancer 7, 19, 20. Our data showed that serum stathmin‐1 can achieve a sensitivity and a specificity of 88.6% and 80.6% in ESCC, respectively, suggesting that stathmin‐1 is a potential serological diagnostic marker. However, those studies are all based on ELISAs. ELISA is not suitable for clinical diagnostic application because of its low throughput, poor stability, and high sample cost, and it is easy to introduce operational errors during the multistep washing and complex operation procedure.

Amplified luminescent proximity homogeneous assay‐linked immunosorbent assay (AlphaLISA) is based on the principle of interaction between biomolecules, along with fluorescence resonance energy transfer luminescence technology. It originated from the light‐initiated chemiluminescent assay (LiCA), which relies on the singlet oxygen energy transfer 21. Upon irradiation with a 680 nm laser, an excitation light of 615 nm is generated. The technology eliminates the need for washing, is fit for automation and mechanization, and has high sensitivity and high throughput characteristics 22. It has been widely used in biomedical research and has become a research hot spot in recent years 22, 23, 24, 25, 26, 27.

Therefore, we developed an AlphaLISA for serum stathmin‐1 detection and systematically evaluated the concentrations of stathmin‐1 in 931 serum samples from healthy individuals and patients with various cancers. In addition, as a cytoplasmic protein lacking the signal peptide, it remains unknown how stathmin‐1 entered into the serum. In this study, we also explore the secretion pathway of stathmin‐1.

## Materials and Methods

### Patients and control

All of the patients were recruited in the Cancer Hospital, Chinese Academy of Medical Sciences from August 2001 to May 2014, including patients with ESCC (*n *=* *364), hepatocellular carcinoma (HCC, *n *=* *53), gastric cancer (GC, *n *=* *59), colorectal cancer (CRC, *n *=* *57), head and neck squamous cell carcinoma (HNSCC, *n *=* *48), lung adenocarcinoma (LAC, *n *=* *53), lung squamous cell carcinoma (LSCC, *n *=* *52), and other types of lung cancer (small cell lung cancer, large cell lung cancer, and lung sarcoma, *n *=* *16). All patients were pathologically diagnosed by two senior pathologists, and serum sample was collected prior to surgical operations or chemo/radiotherapy. Meanwhile, 229 serum samples from healthy individuals (male/female: 178/51; median age, 60 ± 11.2 SD; range 30–82 years) were obtained from the health medical center of the Cancer Hospital, Dongzhimen Hospital, Meitan General Hospital, and Navy General Hospital from February 2003 to November 2013. The enrollment criteria for control subjects were as follows: (1) the absence of benign or malignant tumors and (2) a qualified physical examination finding no dysfunction of vital organs. Serum was separated on ice after rapid centrifugation, and samples were stored in a −80°C refrigerator. The study was approved by the Ethics Committee of the Cancer Hospital, Chinese Academy of Medical Sciences (Beijing, China).

### Preparation of recombinant protein and biotinylation

The coding sequence of *STMN1* was cloned by PCR and inserted into the pET30a vector (EMD Millipore, Burlington, MA). The recombinant stathmin‐1 protein (rSTMN) was expressed by BL21 component cells transformed with pET30a‐STMN1 plasmid and induced by 0.1 mmol/L IPTG. After bacterial lysis, the supernatant was purified by a GE His trap HP column (Little Chalfont, UK). Biotin was labeled with rSTMN by EZ‐Link^™^ Sulfo‐NHS‐SS‐Biotin (Thermo Fisher, Waltham, MA) to enable binding to AlphaLISA streptavidin donor beads.

### High‐affinity monoclonal anti‐stathmin‐1 antibody preparation

With reference to the structure of stathmin‐1, proper epitopes were predicted using Bepitope software 28. To enhance the immunogenicity, the antigenic polypeptide was conjugated with the protein carrier KLH and used to immunize female BALB/c mice (8–12 weeks), which were purchased from Beijng Huafukang Biosciences Co. Inc. (Beijing, China). After the last immunization, the antiserum was screened by ELISA. Mice whose antiserum titers were >10K were chosen for fusion with spleen cells to produce hybridoma cells. Three antibodies with the highest affinity, 3P9, 1B16‐B, and 3B19‐B, were chosen by ELISA for subsequent study.

### Antigen titer analysis

The rSTMN protein was diluted in 50 mmol/L carbonate coating buffer (pH 9.6) to 1, 5, 10, 50, 100, 500, and 1000 ng/mL, and used as coating antigens. Subsequently, 100 μL of 3P9, 1B16‐B, 3B19‐B or commercial rabbit anti‐stathmin‐1 antibody (Cat. No. #ab52630; Abcam, Cambridge, MA) diluted to 100 ng/mL was added to each well and incubated at 37°C for 2 h. After washing three times with 50 mmol/L Tris–HCl buffer containing 0.05% Tween‐20, 1:5000 diluted anti‐mouse/rabbit HRP‐labeled secondary antibody was supplemented and incubated at 37°C for 30 min. After washing five times, the TMB substrate was incubated, and the reaction was terminated. The absorbance value at OD 450 nm was read by a BioRad Model 680 microplate reader (Hercules, CA).

### Western blot analysis

Western blotting was performed as described before 29. In addition to our in‐house antibodies, the relevant antibodies used in this study included anti‐HSP70, anti‐CD63, and anti‐CD9 antibodies (Santa Cruz Biotech., Santa Cruz, CA), anti‐β‐actin (Abmart Co., Ltd., Shanghai, China), and HRP‐conjugated goat anti‐mouse and anti‐rabbit IgG secondary antibodies (Jackson ImmunoResearch Labs, West Grove, PA).The protein bands were visualized using SuperSignal West Femto Maximum Sensitivity Substrate (Thermo Fisher Scientific, Waltham, MA) with the ImageQuant LAS4000 mini system (GE healthcare).

### Establishment of a competitive AlphaLISA

The quantitation of stathmin‐1 was developed based on a homogeneous competitive AlphaLISA system (PerkinElmer Inc., Waltham, MA). The best performing antibody in the titer analysis, 3P9, was further titered with gradually diluted biotinylated rSTMN antigen to determine the optimal antigen‐antibody concentrations that showed the highest AlphaLISA 615 nm emission signal.

All reagents in this assay were diluted in PerkinElmer universal buffer, and two‐step assay procedures were used in 384‐well plates. First, 10 μL samples (serum or standards), 10 μL of 25 ng/mL biotinylated rSTMN, and 10 μL of 156 ng/mL 3P9 antibody were mixed and captured by 10 μL of 100 μg/mL AlphaLISA Protein A‐Acceptor beads. The mixture was incubated at 37°C for 60 min in the dark. Subsequently, 10 μL of 100 μg/mL donor beads was then added, and the plate was incubated at 37°C in the dark for another 30 min. The 615 nm excitation light with the Alpha option was measured by the Enspire^™^2300 multifunctional microplate reader (PerkinElmer).

For calibration, the unlabeled rSTMN was used as the standard for the reference curve. The desired standard concentrations were 0.01, 0.1, 0.5, 1, 5, 10, 50, and 100 ng/mL. Each point was repeatedly measured 20 times. The serum samples were tested in duplicate, and the mean values of AlphaLISA signals were used to calculate the serum stathmin‐1 concentration. Quality controls were included in each plate.

### Performance evaluation of the AlphaLISA

Measurements of 0, 0.1, 10, and 30 ng/mL rSTMN were repeated 20 times in parallel. The recovery rate of our AlphaLISA was calculated by the ratio of the detected value to the expected value. The standard deviation among parallel tests was used to determine the precision of the test.

We also spiked different concentrations of rSTMN into three clinical samples to calculate the sample spike recovery rate. In addition, the lower limit of detection (LOD) is the corresponding concentration of the AlphaLISA signal value for a blank sample plus 3× the standard deviation of the calibration curve.

For comparison, the stathmin‐1 ELISA Quantitative Kit from Cloud‐Clone Corporation (Cat. No. SEC892Hu; Wuhan, China) was also evaluated.

### Cell culture, exosome extraction, and morphology observation

The ESCC cell lines KYSE 170 and KYSE 510 were gifted by Dr. Shimada Y (Kyoto University, Japan).The colorectal cancer cell line HCT 116 and immortalized normal squamous esophagus epithelial cell line Het‐1A were obtained from the American Typical Culture Collection (Rockville, MD). HCT 116‐*TP53* (−/−) cells was gifted by Dr. Bert Vogelstein (John Hopkins University, Baltimore, MD). KYSE 170 overexpressed stathmin‐1 (KYSE 170‐STMN1) and HCT 116‐*p53* MUTANT (R273H) cells were previously constructed by our laboratory 7, 29. Exosome isolation, transmission electron microscopy, and size distribution analysis were all described previously 29.

### Statistical analysis

To compare different groups, Kruskal–Wallis one‐way analysis of variance or Mann–Whitney rank tests were applied. *P*‐values <0.05 were considered statistically significant. Statistical analysis was performed using SPSS v18.0 (IBM software, Chicago, IL).

## Results

### Construction and screening of high‐affinity anti‐stathmin‐1 monoclonal antibodies

**Figure 1:**
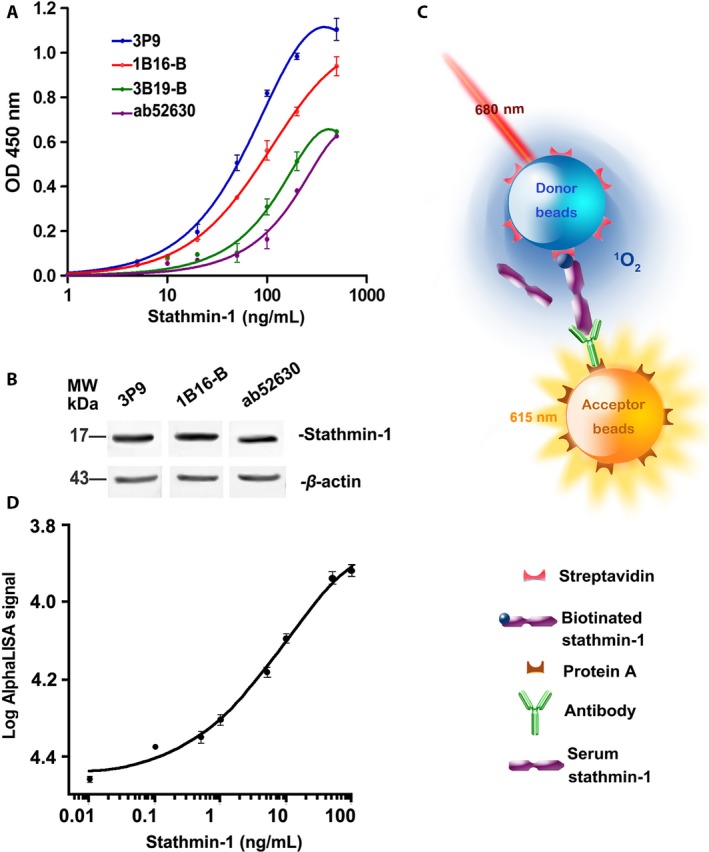
Establishment of a competitive AlphaLISA immunoassay for rapid quantitation of serum stathmin‐1. (A) Antibody titration experiments of the constructed antibodies (3P9, 1B16‐B, and 3B19‐B) and a commercial antibody (ab52630). (B) Western blot analysis of esophageal cancer KYSE 170‐STMN cell lysis for detecting antibody specificity. β‐Actin was used as the internal control. (C) The schematic illustration of our competitive AlphaLISA for serum stathmin‐1. (D) Standard curve of our competitive AlphaLISA immunoassay based on an optimal monoclonal antibody (3P9). Each point was measured with 20 replicates.

Seven epitopes were selected for antibody production (Fig. S1). After two rounds of subclone screening, 17 strains of qualified monoclonal antibody hybridoma cells were selected, which were from two antigenic epitopes (Table S1). The subsequent antigen titer assay and Western blot analysis showed that antibody 3P9, which recognized the C‐terminus of stathmin‐1, had the highest affinity and specificity compared with the other clones and commercial antibodies (Fig. 1A and 1B). Therefore, 3P9 was selected to construct the AlphaLISA.

### Establishment of a competitive AlphaLISA to detect serum stathmin‐1

To determine the optimal concentrations of rSTMN protein and 3P9 antibody for use in the competitive AlphaLISA, the antigen‐antibody titer analysis was performed. The final concentrations of 5 ng/mL rSTMN and 31.25 ng/mL 3P9 antibody in the 50 μL reaction system showed the highest AlphaLISA signal value (Table S2). Therefore, in our subsequent assays, the working concentrations of rSTMN and 3P9 antibody were fixed to 25 and 156 ng/mL, respectively. In this system, biotinylated rSTMN1 protein competed with natural stathmin‐1 in the samples to bind against the anti‐stathmin‐1 antibody, which forms the basis for the competing AlphaLISA (Fig. 1C). The standard curve was produced by 20 repeated measurements of a series of diluted standards (Fig. 1D), revealing a linear dynamic range from approximately 0.1–100 ng/mL, which spanned approximately 2–3 logarithmic units.

### Serum stathmin‐1 detected by AlphaLISA in healthy controls and ESCC patients

**Figure 2:**
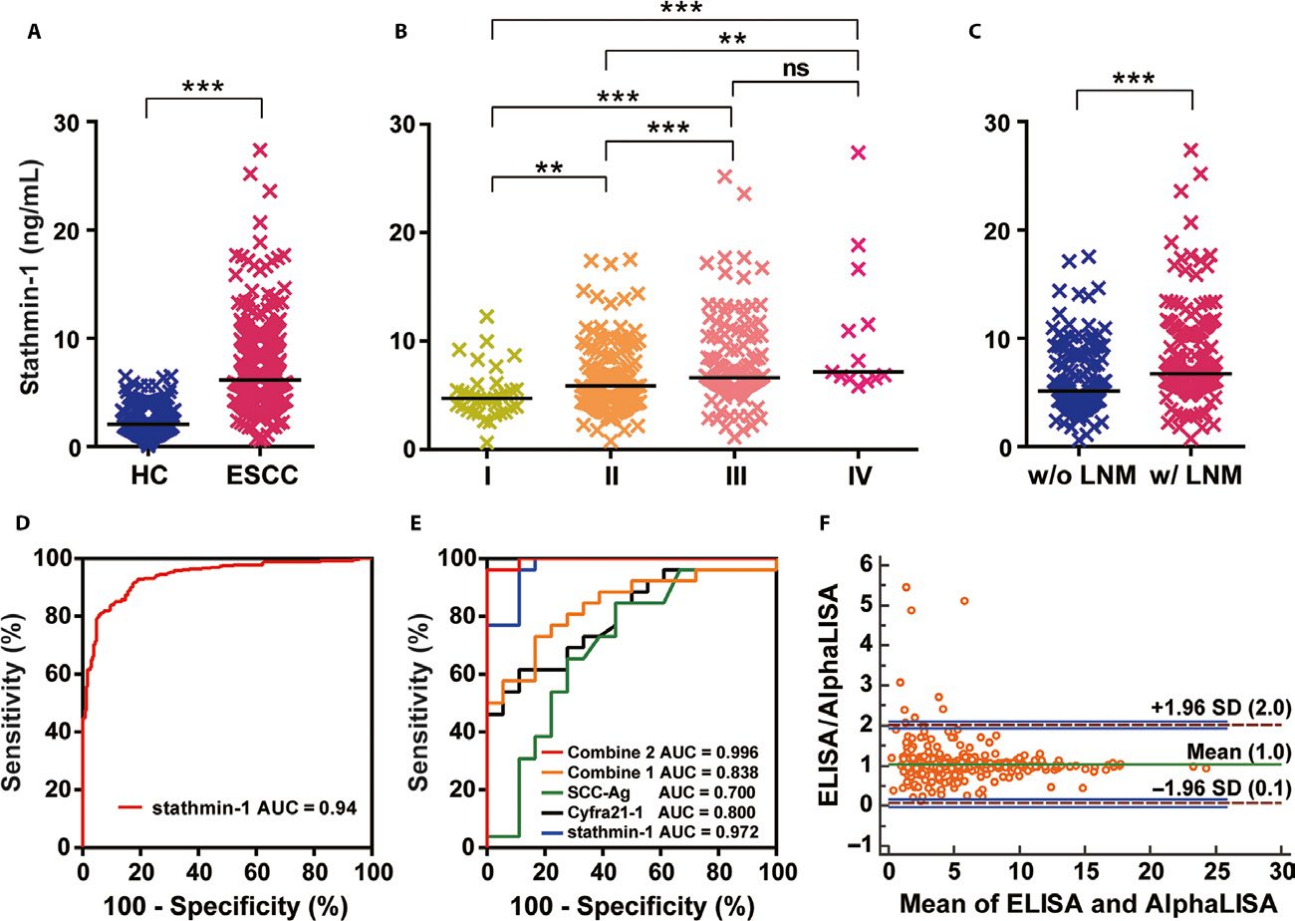
Serum stathmin‐1 was significantly overexpressed in ESCC. (A) Serum levels of stathmin‐1 in healthy controls (HC, *n *=* *229) and patients with ESCC (*n *=* *364). (B) Serologic concentration distribution of stathmin‐1 in ESCC patients with different AJCC stages (I–IV). (C) Serum stathmin‐1 levels were associated with lymph node metastasis in ESCC patients. w/o LNM, without lymph node metastasis; w/LNM, lymph node metastasis. For (A–C), *P <* *0.05; **P <* *0.01; ***P <* *0.001. The median values in each group are shown as black lines. (D) ROC curve of serum stathmin‐1 in healthy controls (*n *=* *229) and patients with ESCC (*n *=* *364). (E) ROC curves of serum stathmin‐1 detected by AlphaLISA (blue), SCC‐Ag (green), Cyfra21‐1 (black),the combination of SCC‐Ag and Cyfra21‐1(combine 1, orange), and the combination of stathmin‐1, SCC‐Ag, and Cyfra21‐1 (combine 2, red). (F) The Bland–Altman plot shows the serum stathmin‐1 levels measured by our AlphaLISA compared with those from commercial ELISA kit. The horizontal axis shows the mean concentrations obtained using the two methods, and the vertical axis shows the difference between the methods. The green line shows the mean differences, dashed purple horizontal lines indicate the 95% limits of agreement (1.96 SD), and the blue lines show the confidence intervals.

The serum stathmin‐1 levels were measured from 229 healthy controls and 364 ESCC patients using our newly established AlphaLISA. The results showed that the median concentrations of stathmin‐1 in healthy controls and ESCC patients were 2.08 ng/mL (range 0.06–6.50 ng/mL) and 6.16 ng/mL (0.60–27.39 ng/mL), respectively (Table 1). The stathmin‐1 levels were significantly increased in ESCC patients (*P *<* *0.0001; Fig. 2A).

**Table 1:** Clinical significance of serum stathmin‐1 in healthy controls and ESCC patients

	HC	Stathmin‐1 levels (ng/mL)		*P* value	ESCC	Stathmin‐1 levels (ng/mL)		*P* value
		Median	Range			Median	Range	
Age (years)								
<60	108	2.08	0.44–6.50	0.1220	166	6.27	0.61–27.39	0.3937
≥60	121	2.20	0.06–6.36		198	6.11	0.74–25.19	
Gender								
Male	178	2.08	0.06–6.50	0.7663	299	6.30	0.74–27.39	0.0210
Female	51	2.07	0.25–5.65		65	5.52	0.61–25.20	
Differentiation grade								
High					73	6.13	0.77–23.59	0.6626
Moderate					165	6.24	0.60–27.39	
Poor					73	6.01	2.03–16.73	
Tumor size (cm)								
≤5					132	6.02	0.61–18.87	0.13695
>5					116	6.40	2.03–23.31	
AJCC staging								
I					35	4.72	0.60–12.27	<0.0001
II					134	5.85	0.77–17.54	
III					127	6.62	1.11–25.20	
IV					13	7.15	5.82–27.39	
Lymph node metastasis								
No					151	5.14	0.60–17.54	<0.0001
Yes					179	6.75	0.77–27.39	

Moreover, serum stathmin‐1 concentrations gradually increased with tumor stage (*P *<* *0.0001; Table 1, Fig. 2B). The median levels of serum stathmin‐1 in stages I, II, III, and IV were 4.72 (0.60–12.27) ng/mL, 5.85 (0.77–17.54) ng/mL, 6.62 (1.11–25.20) ng/mL, and 7.15 (5.82–27.39) ng/mL, respectively. In addition, patients with lymph node metastasis had significantly higher levels of serum stathmin‐1 compared with those without lymph node metastasis (*P *<* *0.0001; Table 1, Fig. 2C). The median concentrations in patients with and without lymph node metastasis were 6.75 (0.77–27.39) ng/mL and 5.14 (0.60–17.54) ng/mL, respectively.

The sensitivity and specificity of serum stathmin‐1 were further evaluated by ROC curves. The area under the ROC curve (AUC) was 0.94(95% CI: 0.92–0.96). In this instance, the optimal stathmin‐1 cutoff value was 4.47 ng/mL, which led to a sensitivity and a specificity of 81.0% (95% CI: 76.6–84.9%) and 93.9% (95% CI: 90.0–96.6%), respectively (Fig. 2D). Thus, stathmin‐1 is an excellent discriminative marker for the diagnosis of ESCC.

### The comparison of the serum Cyfra 21‐1 and SCC‐Ag levels with stathmin‐1

In all of the samples analyzed by AlphaLISA, serum Cyfra 21‐1 and SCC‐Ag were also measured (in 26 ESCC patients and 18 healthy controls).The AUCs were 0.80 (95% CI: 0.67–0.93) for Cyfra 21‐1, 0.70 (95% CI: 0.53–0.87) for SCC‐Ag and 0.84 (95% CI: 0.72–0.96) for a combination of SCC‐Ag and Cyfra 21‐1 (combine 1; Fig. 2E). In comparison, for the limited sample, the AUC was 0.97 (95% CI: 0.93–1.01) for stathmin‐1, and the combination of stathmin‐1, Cyfra 21‐1, and SCC‐Ag achieved an AUC of 1.00 (95% CI: 0.98–1.00; combine 2; Fig. 2E). This result suggested that stathmin‐1 had a significantly superior diagnostic capability compared with Cyfra 21‐1 and SCC‐Ag.

### Performance comparison of the AlphaLISA and ELISAs

The recovery rate, precision, and spike recovery rate of our AlphaLISA were in the range of 100–103.2%, 4.6–7.6%, and 99–104%, respectively (Tables 2 and 3), revealing good accuracy. In addition, the LOD was 0.011 ng/mL, and the time cost was 2 h (Table 4). As shown in Figure 1D, the linear range was approximately 0.10–100 ng/mL. In comparison, the commercial stathmin‐1 ELISA used in our previous study showed an LOD of 0.063 ng/mL and a linear range of 0.156–10 ng/mL (Table 4). The time and sample costs were also much higher than those for our newly established AlphaLISA.

**Table 2:** Recovery rate and precision of AlphaLISA

Stathmin‐1 (ng/mL)	Detected concentration (ng/mL)	Recovery rate (%)	Precision (%)
30.00	30.95	103.2	7.6
10.00	10.24	102.5	7.0
0.10	0.10	100.1	4.6

**Table 3:** Spike recovery rate of AlphaLISA

Sample number	Stathmin‐1 (ng/mL)	Spiked concentration (ng/mL)	Detected concentration (ng/mL)	Spike recovery rate (%)
1	2.05	1.00	3.04	99.1
2	5.63	5.00	10.81	103.4
3	13.27	30.00	44.49	104.1

**Table 4:** Performance comparison of our AlphaLISA and ELISAs

Compared items	ELISA	AlphaLISA
LOD (ng/mL)	0.063	0.011
Time (h)	4.5	2.0
Liner range (ng/mL)	0.156–10	0.10–100
Throughput	Low	High
Sample cost (μL)	50~100	10

Notably, a total of 370 cases, including 100 healthy controls and 270 ESCC patients, were analyzed by both the AlphaLISA and commercial ELISA kit for stathmin‐1. The Pearson correlation coefficient between the two measurements was 0.91 (Fig. S2). A Bland–Altman analysis showed negligible average differences between ELISA and AlphaLISA and an agreement range from 0.1‐fold to twofold change (Fig. 2F). There were a total of nine samples for which the stathmin‐1 concentrations measured by ELISA were twofold higher than those measured by AlphaLISA. Importantly, the difference tended to change with concentration, becoming lower when the concentration was higher, suggesting that it strongly depended on the sensitivity of the measurements. This might result from the narrower linear range and higher LOD of ELISA measurement. Together, our newly established AlphaLISA showed superior accuracy with lower LOD, wider linear range, less sample consumption, and higher throughput.

### Stathmin‐1 is a potential marker for early stage ESCC

**Figure 3:**
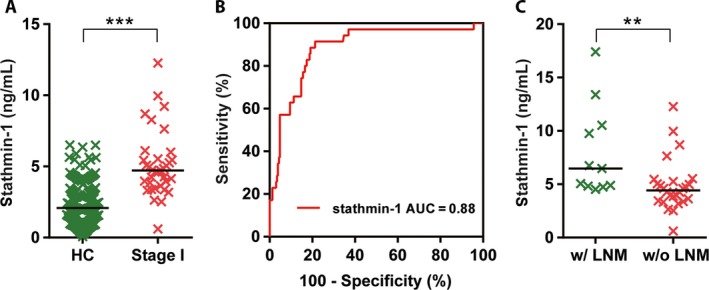
Serum stathmin‐1 was a potential diagnostic marker of early stage ESCC. (A) Serum stathmin‐1 was significantly increased in the early stage of ESCC. (B) ROC curve of stathmin‐1 in healthy controls and patients with early stage ESCC. (C) Serum stathmin‐1 differed between T1 substaging patients without lymph node metastasis and those with lymph node metastasis. w/o LNM, without lymph node metastasis; w/LNM, lymph node metastasis. **P <* *0.01, ***P <* *0.001.

Furthermore, we compared the discriminating capability of serum stathmin‐1 for stage I ESCC patients. The results showed that the serum levels of stathmin‐1 were significantly higher in the early stage of ESCC compared with healthy controls (*P *<* *0.0001; Fig. 3A). The AUC for early stage ESCC reached 0.88 (95% CI: 0.82–0.95). When the threshold was set to 4.47 ng/mL, 57.1% (95% CI: 39.4–73.7%) of early stage patients could be diagnosed as disease with positive expression of serum stathmin‐1 (Fig. 3B).Moreover, the sensitivity in patients with stages II, III, and IV ESCC were 74.6%, 90.5%, and 100%, respectively, with 93.9% specificity.

Notably, for the patients whose tumors invaded only the lamina propria, muscular is mucosae, or submucosa (T1 substaging), the cases who had lymph node metastasis showed higher levels of stathmin‐1 than those without lymph node metastasis (Fig. 3C). The above results suggested that stathmin‐1 can serve as an early diagnostic marker for ESCC and a risk assessment marker for lymph node metastasis.

### Stathmin‐1 is a discriminative biomarker for squamous cell carcinomas

**Figure 4:**
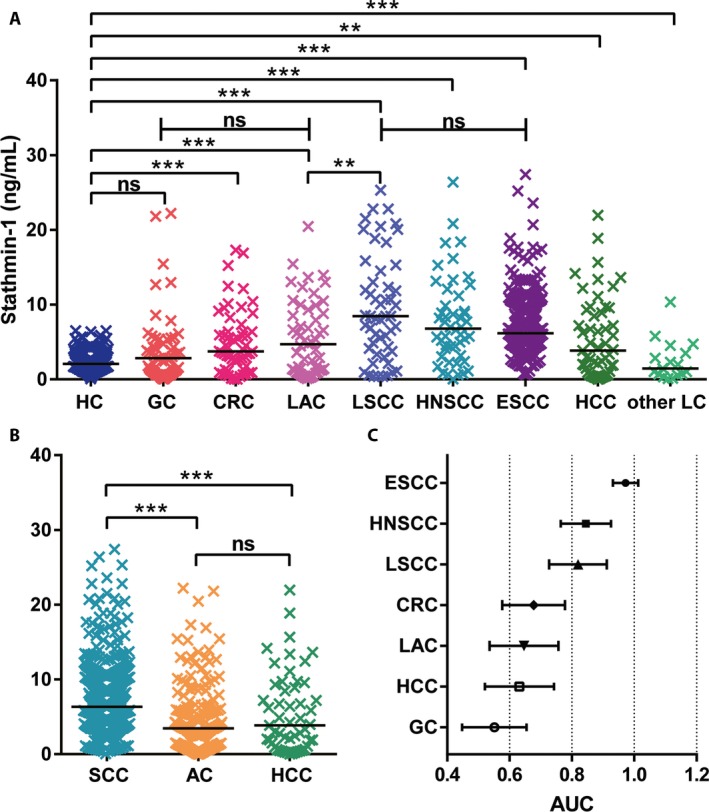
Serum levels of stathmin‐1 were significantly increased in patients with squamous cell carcinomas. (A) Serologic concentration distribution of stathmin‐1 in healthy controls (HC, *n *=* *229) and patients with gastric cancer (GC, *n *=* *59), colorectal cancer (CRC, *n *=* *57), lung adenocarcinoma (LAC, *n *=* *53), lung squamous cell carcinoma (LSCC, *n *=* *52), head and neck squamous cell carcinoma cancer (HNSCC, *n *=* *48), ESCC (*n *=* *364), hepatocellular carcinoma (HCC, *n *=* *53), and other types of lung cancer (other LC, *n *=* *16). (B) Serum levels of stathmin‐1 in squamous cell carcinoma (*n *=* *464), adenocarcinoma (*n *=* *169), and HCC. For (A–B), **P <* *0.01; ***P <* *0.001; ns, not significant. (C) Forest plot of AUC values for stathmin‐1 that distinguished healthy controls from various types of cancer.

Furthermore, the serum levels of stathmin‐1 were measured in other patients with HNSCC, CRC, GC, HCC, and lung cancer. The results showed that the levels of stathmin‐1 were not changed in patients with GC and other types of lung cancer compared with healthy controls, whereas its concentrations were significantly increased in the other tumors (all *P *<* *0.001, Fig. 4A). Notably, there were no significant differences among different types of adenocarcinoma (CRC, GC, and LAC) or squamous cell carcinoma (ESCC, HNSCC, and LSCC) (both *P *>* *0.05). Even for adenocarcinoma and squamous cell carcinoma, which originate from the same organ, the patients with LSCC showed higher serological levels of stathmin‐1 than those with LAC (*P *=* *0.0031).

Importantly, in our analyzed samples, serum stathmin‐1 concentrations were dramatically higher in patients with squamous cell carcinoma than those with adenocarcinoma and HCC (*P *<* *0.001; Fig. 4B). Moreover, there was no significant difference between adenocarcinoma and HCC. When the diagnostic threshold was set to 4.47 ng/mL, the positive rates of stathmin‐1 were 81.0% (295/364), 68.8% (33/48), 71.2% (37/52), 50.9% (27/53), 27.1% (16/59), 43.9% (25/57), and 45.3% (24/53) for ESCC, HNSCC, LSCC, LAC, GC, CRC, and HCC, respectively. The total positive rates of stathmin‐1 for SCC and adenocarcinoma were 78.7% (365/464) and 40.2% (68/169), respectively.

Furthermore, we compared the AUC values among various cancers. As shown in Figure 4C, stathmin‐1 showed superior diagnostic performance for squamous cell carcinoma (AUC > 0.8), whereas the AUC values were lower than 0.7 for the other types of cancer. Thus, these results suggest that stathmin‐1 may serve as a discriminating serological marker for squamous cell carcinoma.

### Stathmin‐1 can be secreted into peripheral blood through the exosome pathway

**Figure 5:**
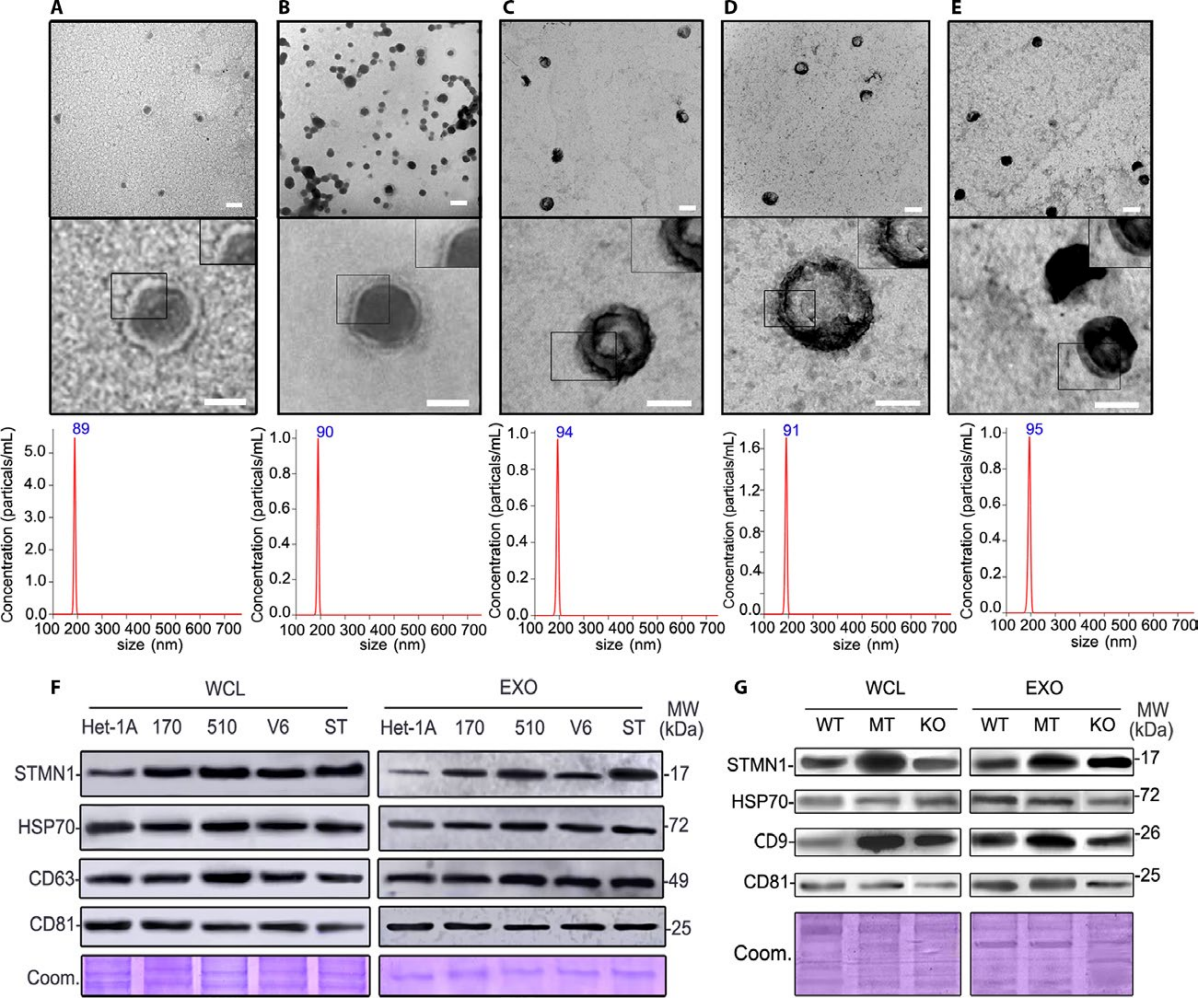
Stathmin‐1 can be secreted via exosomes. Electron micrograph and NanoSight particle‐tracking analysis of exosomes isolated from Het‐1A (A), KYSE 170‐STMN (B), KYSE 170‐pCMV6 (C), KYSE 510 (D), and KYSE170 (E). Scale bars in upper panels and lower panels are 100 nm and 50 nm, respectively. Western blot analysis of stathmin‐1 (STMN1) and exosome markers (HSP70, CD9, CD63, and CD81) in normal squamous esophagus epithelial cells Het‐1A and various esophageal cancer cells (F) as well as colorectal cancer cells (G). WCL: whole cell lysis; EXO: exosome lysis; MW: molecular weight. 170, 510, V6 and ST: esophageal cancer cells KYSE 170, KYSE 510, KYSE 170‐pCMV6, and KYSE 170‐STMN1. WT, MT, and KO: wild type, *TP53* mutant and *Tp53* knockout HCT 116 colorectal cancer cells. Coom: Coomassie blue‐stained protein loading controls.

As a cytoplasmic protein without a signal peptide, it is unclear how stathmin‐1 entered the blood. We further extracted exosomes from an immortalized normal squamous esophagus epithelial cell line (Het‐1A) as well as several ESCC and colorectal cancer cells. Transmission electron microscopy, NanoSight analysis, and Western blot were used to confirm the successful isolation of exosomes with acceptable quality in terms of morphology, size range, and exosomal protein markers (Fig. 5). Importantly, expression of stathmin‐1 was detected in the exosomes of all of cells (Fig. 5F and G). Moreover, expression of stathmin‐1 in exosomes had a similar tendency with that in whole cell lysates. Meanwhile, tumor‐derived exosomes contained higher amount of stathmin‐1 compared with those from Het‐1A, indicating that exosomal stathmin‐1 is a surrogate of its intracellular levels. It is therefore directly confirmed that stathmin‐1 can be secreted into the peripheral blood, where it can act as a tumor marker, through a nonclassical secretory pathway.

## Discussion

In this study, we constructed high‐affinity monoclonal antibodies of stathmin‐1 and then developed a competitive AlphaLISA for rapid, accurate quantitation of serum stathmin‐1 based on the homogeneous reaction. Compared to our previous quantitative results determined by commercial ELISA kits, our newly established AlphaLISA reduced the LOD by approximately sixfold and increased the linear range by at least 10‐fold. The whole detection time was also shortened to 2 h. The measurements of nearly 1000 clinical samples showed that serum stathmin‐1 increased dramatically in patients with squamous cell carcinoma compared with healthy controls and the other cancer types. In ESCC, the sensitivity and specificity reached 81% and 94%, respectively. Importantly, stathmin‐1 had an AUC of 0.88 in the detection of early ESCC. Raised concentrations of stathmin‐1 were also associated with lymph node metastasis, even for early stage ESCC. This is so far the largest scale and most systematic study of serum stathmin‐1 measurement in tumors. Additionally, it was also observed that circulating stathmin‐1 was released by tumor cells via exosomes.

Stathmin‐1 contains 149 amino acids and is mainly located in the cytoplasm. The C‐terminal microtubule‐binding domain (residues 41–140), which consists of an α‐helix motif, can interact with the α/β heterodimer of microtubules to form a tight complex to inhibit microtubule polymerization 30. The epitope of our antibodies with high affinity is for amino acid residues 139–149, which are very close to the microtubule‐binding domain, hinting that the protein fragments of stathmin‐1 recognized by these antibodies in circulation might be functional.

Microtubule depolymerization is associated with mitosis, cell migration, and movement. Actually, stathmin‐1 knockdown had been found to cause cell cycle arrest and promote apoptosis in numerous tumors 31. Moreover, we and other previous studies revealed that overexpression of stathmin‐1 was significantly associated with the higher metastatic potential of various malignant cells 6, 7, 14, 17, 32, 33. Thus, it seems that stathmin‐1 contributes to malignant transformation and tumor progression. Increased stathmin‐1 expression has been found to be significantly associated with poor pathological grade, lymph node metastasis, advanced stage and poor prognosis in ESCC, ovarian cancer, prostate cancer, colorectal cancer, gastric cancer, non‐small cell lung cancer, endometrial cancer, oral cancer, bladder cancer, etc. 9, 10, 11, 12, 13, 14, 15, 16, 17, 18. It remains unknown whether serum stathmin‐1 can also reflect the malignant phenotype of tumor cells. Based on the very limited reports, including our own, serum stathmin‐1 levels have also been associated with advanced stage and lymph node metastasis in ESCC, lung cancer, and transitional cell carcinoma of the bladder 7, 19, 20. Therefore, circulating stathmin‐1 is also derived from oncogenic cells, and it is an ideal biomarker for liquid biopsy.

However, at present, it remains unclear how stathmin‐1 enters into the blood. Previous proteomic studies, including ours, had identified stathmin‐1 as a protein component of exosomes from ovarian, prostate, colorectal, and breast cancer cells as well as melanoma cells 29, 34, 35, 36. Therefore, we hypothesized that stathmin‐1 could be secreted by a noncanonical pathway, such as exosomes, to access the cell culture supernatant or the peripheral blood. Indeed, we confirmed that stathmin‐1 was present in exosomes using a Western blot analysis. The analysis further indicated that malignant cells, which aberrantly overexpressed stathmin‐1, produce oncogenic exosomes, leading to raised levels of stathmin‐1 in serum.

In addition, it was reported that increased expression of stathmin‐1 was more significant in the tissue and serum of patients with lung adenocarcinoma than in those with lung squamous cell carcinoma. This was inconsistent with our findings. On the one hand, this inconsistency may be due to the very limited sample size (*n *=* *48) of the previous work, and on the other hand, it may result from using different detecting assays. The stathmin‐1 ELISA kit used by the previous work is the same as our reference ELISA kit in the present study. It was noteworthy that all of the measured concentrations in the previous work are lower than 8 ng/mL. As shown in our Bland–Altman plot (Fig. 2F), the lower measurements of the ELISA kit are not accurate because of the narrower linear range and higher LOD.

Notably, serum stathmin‐1 was not increased for all of the tumors that had aberrant high expression of stathmin‐1. In our analyzed samples, the patients with gastric cancer and neuroendocrine neoplasm had levels of serological stathmin‐1 similar to those of healthy individuals. Importantly, it seems that stathmin‐1 is a better serological diagnostic marker for squamous cell carcinoma compared with adenocarcinoma and HCC. This result might be related to the tissue expression levels of stathmin‐1. Specifically, stathmin‐1 was higher in cervical squamous cell carcinoma compared with adenocarcinoma 37. Based on the RNA sequencing data in the Cancer Genome Atlas (TCGA), the mRNA expression of STMN1 was slightly higher in squamous cell carcinoma (Fig. S3). Intriguingly, the performance of stathmin‐1 was significantly higher than that of SCC‐Ag, an accepted biomarker for squamous cell carcinoma, at least in ESCC. These results support the great potential and prospect of stathmin‐1 as a diagnostic and predictive marker for metastasis in the clinic.

Recently, AlphaLISA has rapidly been developed for a wide range of clinical applications because of its high sensitivity, high throughput, and simple operation procedure. Some clinical diagnostic assays have appeared, including hepatitis B surface antigen, HE4, allergic celiac disease, and preeclampsia detection kits, among others 24, 38, 39, 40. Our competitive AlphaLISA showed the optimal performance for serum stathmin‐1 detection. The high correlation coefficient with the ELISA kit indicates its accuracy and reliability. The wider linear range and lower LOD suggest its better detection sensitivity and accuracy. The AlphaLISA has other good merits as well, such as high throughput, less time spent per test, a smaller required sample amount and a simpler operation process, which make our AlphaLISA more suitable for future clinical detection of stathmin‐1.

In summary, we have confirmed that stathmin‐1 is secreted by malignant cells in a nonclassical secretory pathway via exosomes and established a competitive AlphaLISA for serum stathmin‐1 detection, which showed better sensitivity and accuracy than the traditional ELISA method. A large‐scale clinical investigation further found that stathmin‐1 is an outstanding diagnostic and predictive marker for squamous cell carcinoma, especially for ESCC.

## Conflict of Interest

We have no conflict of interest to declare.

## Supporting information


**Figure S1.** The selected epitopes of stathmin‐1 for the construction of monoclonal antibodies.
**Figure S2**. The correlation plot of stathmin‐1 concentrations measured by our newly established AlphaLISA and commercial ELISAs.
**Figure S3**. The median expression level of *STMN1* mRNA presented in representative TCGA RNA sequencing datasets.
**Table S1**. Two predicted specific epitopes and their corresponding 17 hybridoma cell strains.
**Table S2**. The antigen‐antibody titer analysis of 3P9 antibody and biotin‐labeled rSTMN protein.
