# Supplementary material for: A novel rapid quantitative method reveals stathmin‐1 as a promising marker for esophageal squamous cell carcinoma
Source: Cancer Med. 2018 Mar 25;7(5):1802–13. doi: 10.1002/cam4.1449 (PMC5943482; doi:10.1002/cam4.1449)
Supplement: Supplementary file 1 — Figure S1. The selected epitopes of stathmin‐1 for the construction of monoclonal antibodies. Figure S2. The correlation plot of stathmin‐1 concentrations measured by our newly established AlphaLISA and commercial ELISAs. Figure S3. The median expression level of STMN1 mRNA presented in representative TCGA RNA sequencing datasets. Table S1. Two predicted specific epitopes and their corresponding 17 hybridoma cell strains. Table S2. The antigen‐antibody titer analysis of 3P9 antibody and biotin‐labeled rSTMN protein. [file CAM4-7-1802-s001.pdf]

A novel rapid quantitative method reveals stathmin-1 as a promising marker for esophageal squamous cell carcinoma

Lu Yan, Xiu Dong, Jiajia Gao, Fang Liu, Lanping Zhou, Yulin Sun, Xiaohang Zhao

State Key Laboratory of Molecular Oncology, National Cancer Center/Cancer Hospital, Chinese Academy of Medical Sciences & Peking Union Medical College, Beijing, China

Supporting Information

Additional supporting information may be found in the online version of this article:

Figure S1. The selected epitopes of stathmin-1 for the construction of monoclonal antibodies.

Figure S2. The correlation plot of stathmin-1 concentrations measured by our newly established AlphaLISA and commercial ELISA assays.

Figure S3. The median expression level of *STMN1* mRNA presented in representative TCGA RNA sequencing datasets.

Table S1. Two predicted specific epitopes and their corresponding 17 hybridoma cell strains.

Table S2. The antigen-antibody titer analysis of 3P9 antibody and biotin-labeled rSTMN protein.

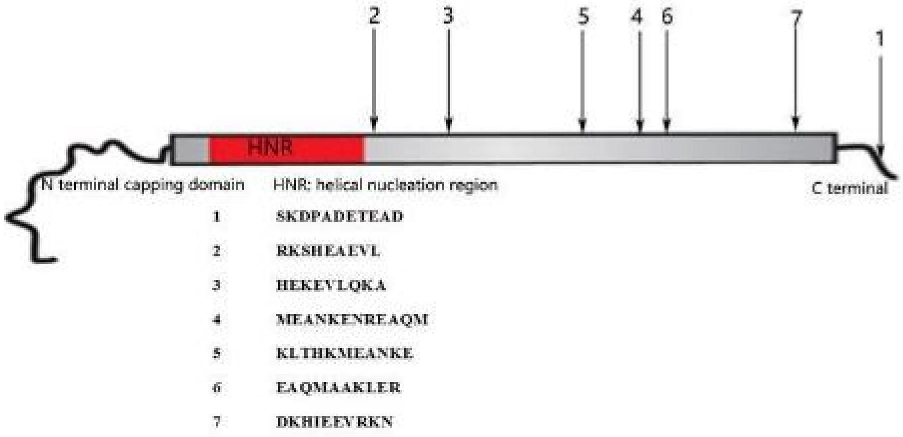

Figure S1. The selected epitopes of stathmin-1 for the construction of monoclonal antibodies.

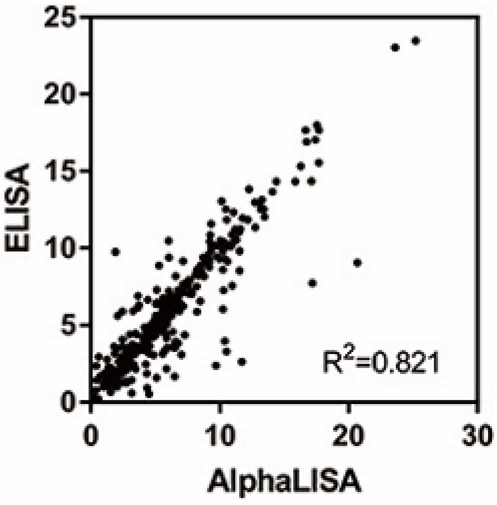

Figure S2. The correlation plot of stathmin-1 concentrations measured by our newly established AlphaLISA and commercial ELISA assays.

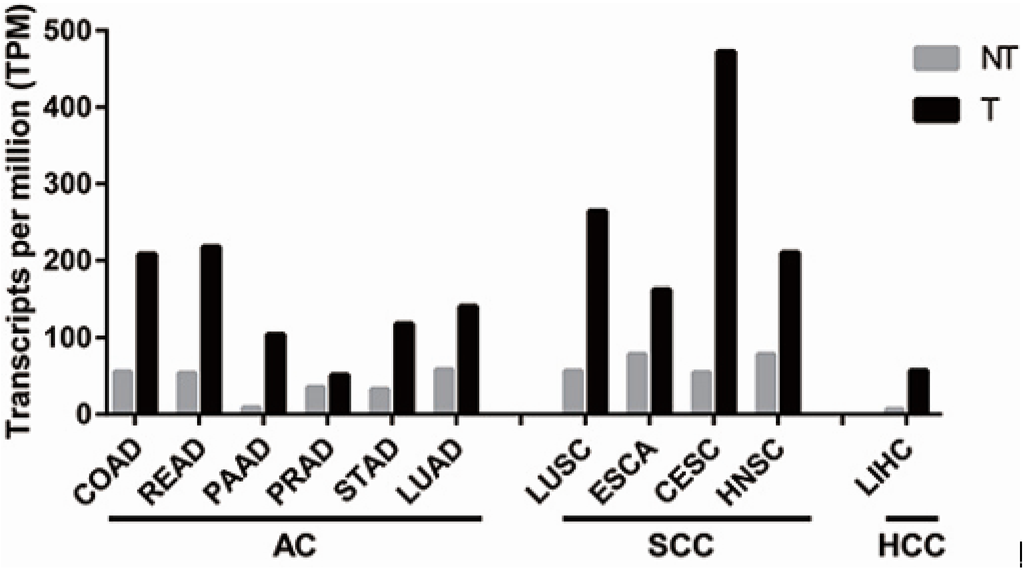

Figure S3. The median expression level of *STMN1* mRNA presented in representative TCGA RNA sequencing datasets. T, tumor tissues; NT, non-tumor tissues; AC, adenocarcinoma; SCC, squamous cell carcinoma; COAD, colon adenocarcinoma; READ, rectum adenocarcinoma; PAAD, pancreatic adenocarcinoma; PRAD, prostate adenocarcinoma; STAD, stomach adenocarcinoma; LUAD, lung adenocarcinoma; LUSC, lung squamous cell carcinoma; ESCA, esophageal carcinoma; CESC, cervical squamous cell carcinoma and endocervical adenocarcinoma; HNSC, head and neck squamous cell carcinoma; LIHC, liver hepatocellular carcinoma. It should be noted that ESCA samples include ESCC and esophageal adenocarcinoma.

Table S1. Two predicted specific epitopes and their corresponding 17 hybridoma cell strains.

Cell strains	Epitopes
2P2-B	SKDPADETEAD
1K15-B	SKDPADETEAD
4C22-B	SKDPADETEAD
3O7	SKDPADETEAD
1M15	SKDPADETEAD
2G19	SKDPADETEAD
3O7-B	SKDPADETEAD
1B16-B	SKDPADETEAD
2L18	SKDPADETEAD
3O20-B	SKDPADETEAD
1M15-B	SKDPADETEAD
4C22	SKDPADETEAD
1B16-D	SKDPADETEAD
3P9	SKDPADETEAD
1B16-C	SKDPADETEAD
3B19-B	KLTHKMEANKE
3B19	KLTHKMEANKE

Table S2. The antigen-antibody titer analysis of 3P9 antibody and biotin-labeled rSTMN protein

Antibody/Antigen(ng/mL)	500	100	50	10	5	1	0.5	0.1
500	9627.5	11719.5	9860.0	8474.0	7540.0	7028.0	7673.5	8823.5
250	13700.0	14643.5	13177.0	12616.0	10338.0	9975.0	7189.0	10267.0
125	13825.5	16410.0	17659.5	13192.0	11564.0	10927.5	7616.5	10499.5
62.5	14902.5	16022.0	18433.0	14909.5	16118.5	16217.0	14415.0	14258.0
31.25	15055.0	16840.0	20658.0	23455.0	28890.0	21402.5	17433.0	10981.0
15.60	12482.0	14570.0	15447.0	18601.0	14146.0	12638.0	13858.0	15349.0
7.80	16733.0	14735.0	13683.0	16282.0	13997.0	12052.0	20807.0	13479.0
3.90	17228.0	16840.0	13553.0	12434.0	10415.0	8510.0	10761.0	9889.5
